# Proline-mediated regulation on jasmonate signals repressed anthocyanin accumulation through the MYB-bHLH-WDR complex in rice under chromium exposure

**DOI:** 10.3389/fpls.2022.953398

**Published:** 2022-08-02

**Authors:** Qing Zhang, Yu-Xi Feng, Peng Tian, Yu-Juan Lin, Xiao-Zhang Yu

**Affiliations:** College of Environmental Science and Engineering, Guilin University of Technology, Guilin, China

**Keywords:** rice, chromium, proline, jasmonic acid, anthocyanin

## Abstract

Toxic metal-induced overaccumulation of anthocyanin (ATH) in plants can oxidize proteins and break DNA. Herein, the role of exogenous proline (Pro) on the repression of ATH accumulation in rice seedlings during hexavalent chromium [Cr(VI)] exposure was studied. Results indicated that exogenous Pro-mediated regulation of jasmonate signals activated the MYB-bHLH-WDR complex to repress ATH accumulation in rice tissues under Cr(VI) stress. Biochemical and transcript analysis indicated that exogenous Pro promoted the synthesis of jasmonic acid (JA) and its molecularly active metabolite jasmonic acid isoleucine (JA-Ile) in rice tissues under Cr(VI) stress. Increment in the endogenous level of jasmonates positively triggered the expression of genes responsible for the JA signaling pathway and activated the MYB-bHLH-WDR complex, eventually repressing the glycosylation of anthocyanidin to form ATH in rice tissues. In conclusion, exogenous proline-mediated regulation on jasmonate signals was tissue-specific under Cr(VI) stress and a more positive effect was detected in shoots rather than roots.

## Introduction

The occurrence of chromium (Cr) contamination in the agricultural system has attained pivotal concern worldwide (Shahid et al., 2017). Rice is the most important staple food in Asia (Awotide et al., 2016). Numerous researches reported that soil Cr contamination results in a remarkable decrease in the yield and quality of rice (Ma et al., 2016; Infante et al., 2021; Xiao et al., 2021). One of the most frequently detected modes of Cr(VI) phytotoxicity is to stimulate over-production of reactive oxygen species (ROS) in plant tissues that destroy the plant’s internal defense system and cause oxidative damage, whereas irreversible modification of proteins, lipid peroxidation, and DNA lesion is detectable, and eventually retards plant growth and even causes cell death (Gomes et al., 2017; Fan et al., 2020; Nie et al., 2021; Zhang et al., 2021).

Mostly, alteration of secondary metabolism is one of the most important survival strategies of plants in response to the imposition of toxic metals (Shitan, 2016), in which plants can use phenylalanine by assimilating this precursor directly into secondary metabolism *via* the phenylpropanoid pathway (Lin et al., 2019). Among other intermediates, anthocyanin (ATH) is the most conspicuous in the family of flavonoids with dual roles *in vivo* during plant growth and development (Ling et al., 2021), most likely depending on its content and subcellular distribution in plant cells. ATH accumulated in vacuole can protect chloroplasts against photo-oxidation and UV irradiation damage (Collin et al., 2008; Park et al., 2011). While excessive accumulation of ATH in cytosol has cytotoxic/genotoxic effects (Zhao et al., 2008), it weakens the yield and quality of crop plants (Ling et al., 2021).

Jasmonates are spontaneously occurring signaling molecules in plants. Among other active metabolites, jasmonic acid (JA) is a vital hormone in plants that serves as a regulatory agent to mediate multiple physiological processes and also functions as a defense signaling molecule to cope with Cr(VI) stress (Michael et al., 2015; Kamran et al., 2021). Numerous studies reported the importance of JA in the regulation of ATH accumulation in plants under various abiotic conditions (Kondo et al., 2021). Additionally, JA-mediated regulation of various transcription factors (TFs) corresponding to different abiotic stresses in plants has been experimentally clarified (Ruan et al., 2019). Other studies suggested that the MYB-bHLH-WDR complex has an important regulatory role to reduce ATH accumulation in plants (Broun, 2005; Qi et al., 2011). Therefore, concerted attempts are needed to clarify the bridge of the MYB-bHLH-WDR complex involved in the interaction between the jasmonate signals and ATH accumulation in plants under Cr(VI) exposure.

Recently, the application of exogenous amino acids has become one of the most effective methods to regulate plant growth and development under environmental abuse. For example, Kan et al. (2017) reported that exogenous glutamate can rapidly induce the expression of genes involved in the metabolism and defense responses in rice plants. Sadak et al. (2012) observed that exogenous arginine can enhance the photosynthesis system and regulate the physiological performance of sunflowers under salt stress. It is intriguing to notice that the function of proline (Pro) in plants is manifold compared with other amino acids, such as osmolyte balancer, free radical scavenger, and macromolecules stabilizer (Zhang et al., 2015, 2021). Previous studies suggested that exogenous Pro positively regulated the enzyme activities of the ascorbate-glutathione cycle in tobacco under salt stress, and minimized the negative effect (Hoque et al., 2007). A decrease in soluble sugar was detected in *Arabidopsis* in response to drought stress in presence of exogenous Pro by lifting the efficiency of the photosystem II system (Moustakas et al., 2011). Exogenous Pro reduced MDA content and root cell viability in Cr(VI)-treated rice seedlings and decreased lipid peroxidation (Yu et al., 2017). Also, crosstalk between Pro and plant hormone (e.g., JA, gibberellins, abscisic acid, cytokinins, and ethylene) in plants under stress conditions has been documented (Alvarez et al., 2022). However, little is known about the Pro-mediated regulation of ATH accumulation through the JA signaling pathway and MYB-bHLH-WDR complex in rice seedlings under Cr(VI) exposure.

Herein, we hypothesized that exogenous Pro-mediated regulation of jasmonate signals initiates the MYB-bHLH-WDR complex to repress ATH accumulation in rice plants in response to Cr(VI) stress. To clarify this hypothesis, the following studies were carried out: (1) to determine the content of 12-oxo-phytodienoic acid (OPDA), JA, jasmonic acid isoleucine (JA-Ile), and ATH in rice plants under Cr(VI) stress, with or without application of exogenous Pro; (2) to quantify the transcript level of JA synthesis, JA signaling pathway, MYB-bHLH-WDR complex, and anthocyanidin synthesis related genes in rice plants under Cr(VI) stress, with or without application of exogenous Pro; (3) to construct the Pro-mediated regulation network involved in jasmonate signals, MYB-bHLH-WDR complex, and anthocyanidin synthesis in rice plants under Cr(VI) stress.

## Materials and methods

### Plant cultivation and chemical preparation

The seeds of rice (*Oryza sativa* L. XZX 45) were obtained from the Hunan Academy of Agricultural Sciences, which were prepared as described in our previous work (Zhang et al., 2021). In brief, rice seeds were soaked for 12 h before cultivation, then placed in clean river sand inside plastic cups, and deployed in an artificial climate chamber to maintain stable running conditions (light: 20,000 lux, temperature: 25°C ± 0.5°C and humidity: 60% ± 2%). The modified 8692 nutrient solution was prepared as per the protocol (Feng et al., 2019). After 16-day pre-growth, seedlings were collected and cleaned with distilled water for the subsequent tests.

Two different treatments were conducted: (1) Cr(VI) treatment: 16-days old seedlings were grown in nutrient solution containing 0, 2.0, 8.0, and 16.0 mg Cr/L for 2-days exposure; (2) “Pro + Cr(VI)” treatment: 16-days old seedlings were first pretreated in Pro solution (1 mM) after soaking for 12 h (Yang et al., 2021), and then transferred into the nutrient solution containing 0, 2.0, 8.0, and 16.0 mg Cr/L for 2-days exposure. Three different effective concentrations of Cr(VI; i.e., EC_20_, EC_50_, and EC_75_) were used based on the inhibition rate with 20%, 50%, and 75% of relative growth rate during 2-days exposure (Zhang et al., 2021). The pH of solution was approximately 6.7 to 6.9 during the entire period of exposure. To lessen water loss and repress the growth of algae, each flask was wrapped with aluminum foil. Four independent replicates were deployed in each experiment, and 10 seedlings of similar size were taken for each replicate (Zhang et al., 2021). The grade of all chemicals used was analytical purity (AR).

### Measurement of jasmonates

After exposing to Cr(VI) solution, the rice tissues were collected and ground with liquid nitrogen with internal standards. The powdery tissue samples were used for the extraction, purification, and analysis of jasmonates. Quantification of jasmonates, including 12-oxo-phytodienoic acid (OPDA), jasmonic acid (JA), and jasmonic acid isoleucine (JA-Ile) was determined by ultra-performance liquid chromatography–tandem mass spectrometry (UPLC-MS/MS). The procedures for extraction, purification, and analysis were described by Shao et al. (2019; Supporting Information M1). Three biological replicates were prepared for each sample.

### Quantification of anthocyanin (ATH)

After freezing with liquid nitrogen, rice tissues were ground and immersed in 5 ml of hydrochloric acid methanol solution (15% hydrochloric acid, 95% methanol = 15:85, v/v), and isolated from light at room temperature (~25°C) for 4 h. After centrifuging at 3,500 g for 5 min at 4°C, the supernatants were collected for ATH measurement by a spectrophotometric method against hydrochloric acid methanol solution as a reference (Ling et al., 2021). Calculation of ATH content in plant tissues was based on the following equation:


(1) $$\begin{array}{l} C_{ATH}\left(nmol/g\right)\\ =\frac{\left[\left(OD_{530}-OD_{620}\right)-0.1\times \left(OD_{650}-OD_{620}\right)\right]}{\varepsilon }\times \frac{V}{M}\times 10^{6} \end{array}$$

Where *OD* is the absorbance at 530, 620, and 650 nm, respectively, *ε* is the molar extinction coefficient of ATH (4.62 × 10^6^), *V* is the total extract volume (ml), and *M* is the weight of fresh samples (g).

### RNA extraction

Total RNA was extracted from rice tissues of the Cr-treated and non-treated seedlings by using an Ultrapure RNA Kit (CWBio, Taizhou, China). DNase I (CWBio, Taizhou, China) was used to clear away genomic DNA contamination in RNA extract. After that, an RNeasy MinElute Cleanup Kit (Qiagen, Hilden, Germany) was selected to purify the total RNA. Four biological replicates were prepared for each sample.

### Gene identification and RT-qPCR analysis

Thirty-eight genes responsible for JA synthesis (Module 1, 16 genes), JA signaling pathway (Module 2, 8 genes), MYB-bHLH-WDR complex (Module 3, 11 genes), and anthocyanidin synthesis (Module 4, 3 genes), respectively, were identified from three rice databases (RGAP, CRTC, and RAP-DB) and assayed for RT-qPCR tests after Cr(VI) exposure. All gene primer sequences are listed in Supplementary Table S1.

RT-qPCR cycling conditions were set up as follows: (1) denaturation at 95°C for 10 s; (2) annealing at 58°C for 30 s; and (3) extension at 72°C for 32 s. This cycle was repeated 40 times. The RT-qPCR analysis was performed by the 7,500 Fast RT-qPCR system (Applied Biosystems) and SYBR green chemistry. The house-keeping gene was *OsGAPDH* (glyceraldehyde-3-phosphate dehydrogenase, LOC_Os08g03290.1; Yang et al., 2021). The relative expression of each targeted gene was calculated by the standard 2^−ΔΔCT^ method (Schmittgen and Livak, 2008).

### Estimation of gene expression variation factors

The gene expression variation factors (*GEVFs*) were used to estimate the effect of exogenous Pro on the expression of genes in rice plants after Cr(VI) exposure, based on the following equation (Zhang et al., 2022):


(2) $$GEVFs=\frac{FC_{\left(\mathrm{Pr}o+Cr\right)}-FC_{\left(Cr\right)}}{FC_{\left(Cr\right)}}\times 100\%$$

Wherein *FC_(Pro + Cr)_* refers to the gene expression from the “Pro + Cr(VI)” treatments; *FC_(Cr)_* refers to the gene expression from the Cr(VI) treatments. The threshold values of *GEVFs* are deployed as >25% (promoting genes) or <−25% (repressing genes). Noted that the “promoting genes” under different ECs of Cr(VI) were discussed in this study.

### Data analysis

The experimental results were given as mean ± SD. The significant difference between the treatments and control was studied by Tukey multiple comparison test at *p* < 0.05. The asterisk (^*^) represents the significant difference between Cr treatments and “Pro + Cr(VI)” treatments. The letter indicates the significant difference between the treated seedlings and the control. Exogenous Pro-mediated gene expression of Modules 1 to 3 in rice seedlings at 0 mg Cr/L treatment (i.e., Control) is shown in Supplementary Figure S1. The Search Tool for the Retrieval of Interacting Genes (STRING) program was used to analyze and visualize the gene interaction network.

## Results

### The content of jasmonates in rice seedlings

**Figure 1:**
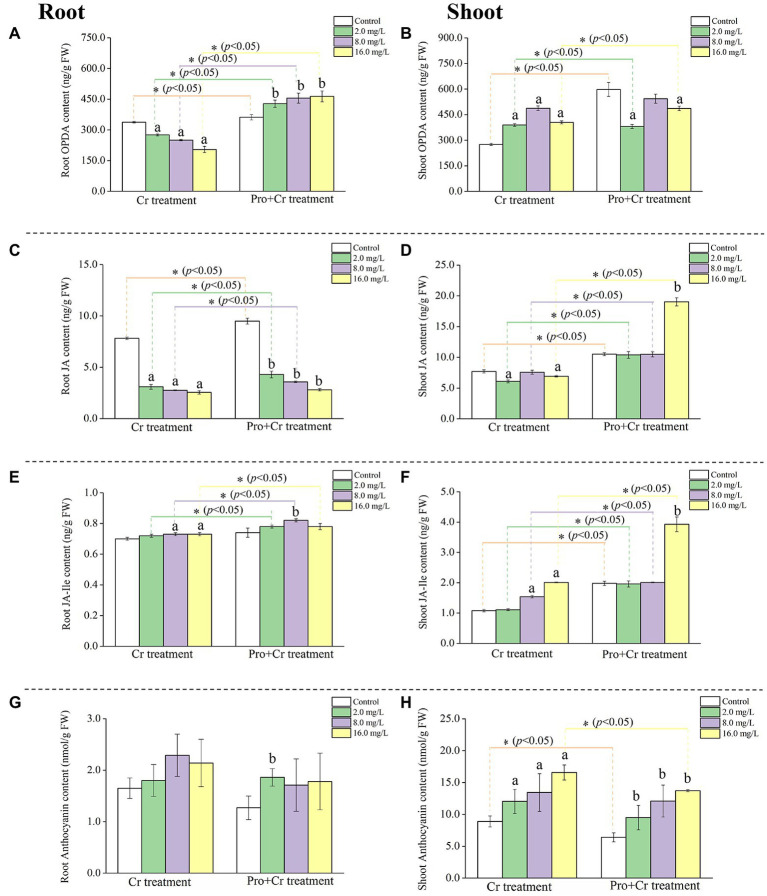
The content of jasmonates and ATH in rice tissues under Cr(VI) stress in the presence or absence of Pro. The content of OPDA in rice roots **(A)** and shoots **(B)**; The content of JA in rice roots **(C)** and shoots **(D)**; The content of JA-Ile in rice roots **(E)** and shoots **(F)**; The content of ATH in rice roots **(G)** and shoot **(H)**. The asterisk (^*^) refers to the significant difference between Cr(VI) treatments and Pro + Cr(VI) treatments. The letter refers to the significant difference between the treated seedlings and the control.

The content of OPDA (ng/g FW) linearly decreased in rice roots with increasing Cr(VI) concentrations (*p* < 0.05), while the content of OPDA showed an inverted “U” curve in rice shoots, with the highest content (487.90 ± 13.59 ng/g FW) at 8.0 mg Cr/L. Exogenous Pro significantly (*p* < 0.05) increased the content of OPDA in rice roots compared to the control, while the content of OPDA generally decreased in rice shoots, with the lowest content (380.45 ± 11.65 ng/g FW) at 2.0 mg Cr/L (Figures 1A,B).

The content of JA remarkably (*p* < 0.05) reduced in root tissues under Cr(VI) stress compared to the control, ranging from 7.82 ± 0.12 to 2.56 ± 0.17 ng/g FW, while a similar decreasing trend was observed in rice shoots. The content of JA significantly (*p* < 0.05) decreased in rice roots from 9.49 ± 0.29 to 2.81 ± 0.11 ng/g FW under “Pro + Cr(VI)” treatments compared to the control. Interestingly, the content of JA significantly (*p* < 0.05) increased in rice shoots at 16.0 mg Cr/L with 19.03 ± 0.66 ng/g FW (Figures 1C,D).

The content of JA-Ile increased very little in rice roots under Cr(VI) stress, ranging from 0.70 ± 0.01 to 0.73 ± 0.01 ng/g FW. Similarly, the content of JA-Ile increased from 1.98 ± 0.07 to 3.93 ± 0.24 ng/g FW in rice shoots under Cr(VI) stress. Under the “Pro + Cr(VI)” treatment, the content of JA-Ile first increased and then decreased in rice roots, with the highest value of 0.82 ± 0.01 ng/g FW at 8.0 mg Cr/L. The content of JA-Ile significantly (*p* < 0.05) increased in rice shoots at 16.0 mg Cr/L with 3.93 ± 0.24 ng/g FW (Figures 1E,F).

It is noticed that the content of OPDA, JA, and JA-Ile in both rice roots and shoots from “Pro + Cr(VI)” treatments was generally higher than that from Cr(VI) treatments, suggesting a positive regulatory role of exogenous Pro on the synthesis of jasmonates-related compounds in rice seedlings under Cr(VI) exposure.

### The content of ATH in rice seedlings

The content of ATH (nmol/g FW) slightly increased in rice roots with enhancing Cr(VI) concentrations, while the content of ATH remarkably (*p* < 0.05) increased in rice shoots with enhancing Cr(VI) concentrations. A similar accumulation pattern of ATH in both rice tissues was observed in the “Pro + Cr(VI)” treatments with increasing Cr(VI) concentration (Figures 1G,H). However, the content of ATH in rice shoots from “Pro + Cr(VI)” treatments was lower (*p* < 0.05) than that from Cr(VI) treatments. This result suggested that exogenous Pro effectively reduced ATH accumulation in rice shoots under Cr(VI) stress.

### Expression of genes responsible for jasmonate signals in rice seedlings

#### Gene expression at EC_20_ of Cr(VI)

**Figure 2:**
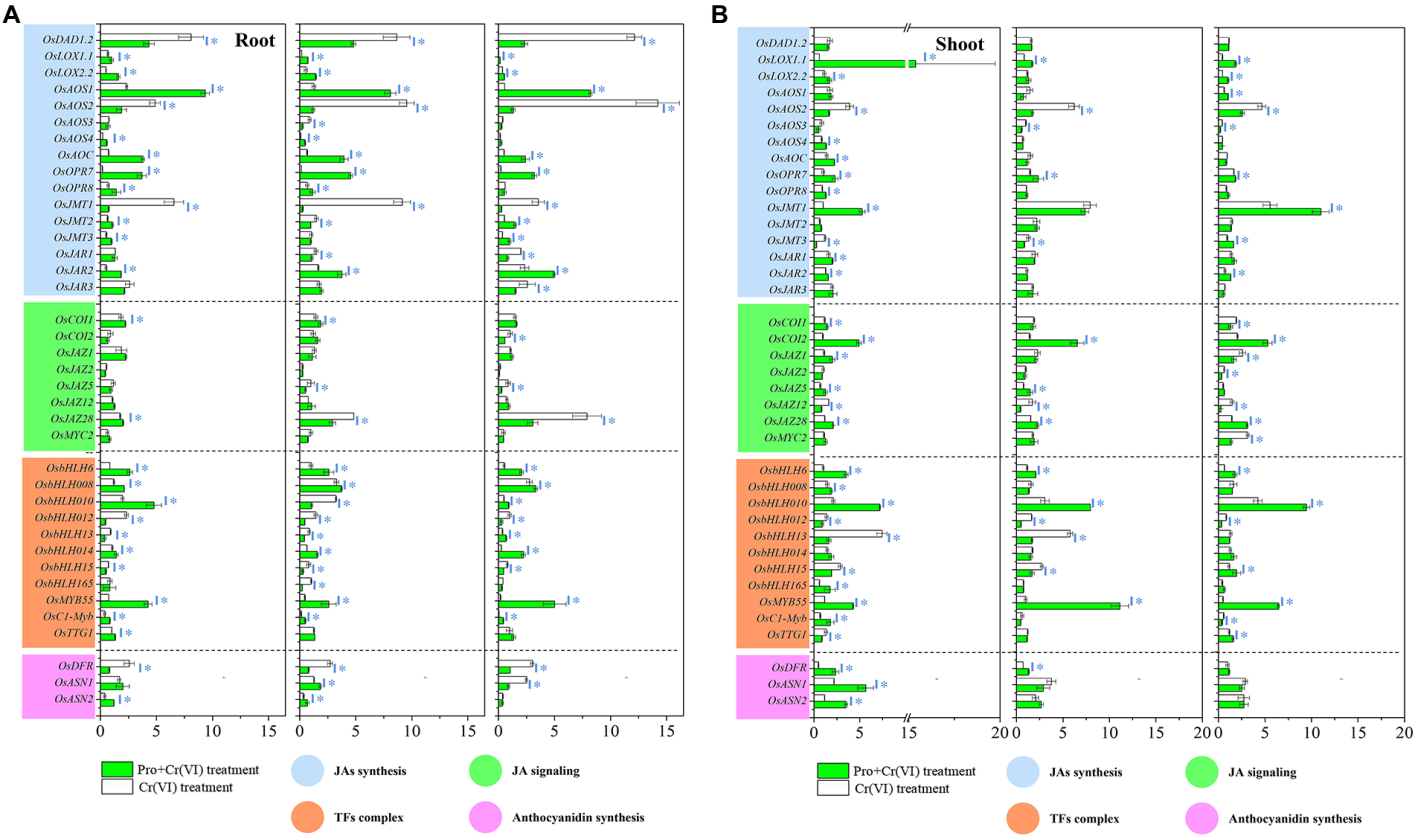
The relative expression of JA synthesis, JA signaling, MYB-bHLH-WDR complex, and anthocyanidin synthesis-related genes in rice seedlings under Cr(VI) stress in the presence or absence of Pro. **(A)** The expression of these genes in rice roots under Cr(VI) stress in the presence or absence of Pro. **(B)** The expression of these genes in rice shoots under Cr stress in the presence or absence of Pro. The relative expression level of control is 1. The asterisk (^*^) refers to the significant difference between Cr(VI) treatments and Pro + Cr(VI) treatments.

In roots of rice seedlings, expression of JA synthesis-related genes, that is, *OsLOX1.1*, *OsLOX2.2*, *OsAOS1*, *OsAOS4*, *OsAOC*, *OsOPR7*, *OsOPR8, OsJMT2*, and *OsJMT3,* from the “Pro + Cr(VI)” treatments were significantly (*p* < 0.05) higher than that of Cr(VI) treatments. Also, expression of genes related to the JA signaling pathway, that is, *OsJAR2*, *OsCOI1*, *OsJAZ12*, and *OsJAZ28,* under “Pro + Cr(VI)” treatments were significantly upregulated (*p* < 0.05), compared with Cr(VI) treatments (Figure 2A).

In shoots of rice seedlings, Pro-mediated upregulation (*p* < 0.05) of JA synthesis-related genes, that is, *OsLOX2.2*, *OsAOS4*, *OsAOC*, *OsOPR7*, *OsOPR8*, and *OsJMT1* was detected, compared with Cr(VI) treatments. Higher expression of genes related to the JA signaling pathway, that is, *OsJAR1*, *OsJAR2*, *OsCOI1*, *OsCOI2*, *OsJAZ1*, *OsJAZ5*, and *OsJAZ28* from the “Pro + Cr(VI)” treatments was also detected (*p* < 0.05), compared with Cr(VI) treatments (Figure 2B).

#### Gene expression at EC_50_ of Cr(VI)

In root tissues, JA synthesis-related genes, that is, *OsLOX1.1* and *OsOPR7* under “Pro + Cr(VI)” treatments were significantly (*p* < 0.05) upregulated, compared with Cr(VI) treatments. Higher expression of genes activated in the JA signaling pathway, that is, *OsJAR2*, *OsCOI1*, and *OsJAZ12,* under “Pro + Cr(VI)” treatments was observed (*p* < 0.05), compared with Cr(VI) treatments (Figure 2A).

In shoot tissues, JA synthesis-related genes, that is, *OsLOX1.1* and *OsOPR7,* under “Pro + Cr(VI)” treatments were significantly (*p* < 0.05) upregulated, compared with Cr(VI) treatments. Higher expression of genes involved in the JA signaling pathway, that is, *OsCOI2*, *OsJAZ5*, and *OsJAZ28,* under “Pro + Cr(VI)” treatments was determined (*p* < 0.05), compared with Cr(VI) treatments (Figure 2B).

#### Genes expression at EC_75_ of Cr(VI)

In root tissues, JA synthesis-related genes, that is, *OsLOX1.1, OsLOX2.2, OsAOS1, OsAOS4, OsAOC, OsOPR7, OsJMT2,* and *OsJMT3,* under “Pro + Cr(VI)” treatments were significantly (*p* < 0.05) upregulated, compared with Cr(VI) treatments. Higher expression of genes related to the JA signaling pathway, that is, *OsJAR2* under “Pro + Cr(VI)” treatments were noticed (*p* < 0.05), compared with Cr(VI) treatments (Figure 2A).

In shoot tissues, JA synthesis-related genes, that is, *OsLOX1.1*, *OsLOX2.2*, *OsAOS1*, *OsOPR7*, *OsOPR8, OsJMT1*, and *OsJMT3,* under “Pro + Cr(VI)” treatments were significantly (*p* < 0.05) upregulated, compared with Cr(VI) treatments. Higher expression of genes involved in the JA signaling pathways, that is, *OsJAR2*, *OsCOI2*, *OsJAZ5*, and *OsJAZ28,* under “Pro + Cr(VI)” treatments were detected (*p* < 0.05), compared with Cr(VI) treatments (Figure 2B).

These results suggested that the expression of jasmonate signals-related genes in rice seedlings is tissue-specific and highly dependent on the Cr(VI) concentrations. Application of exogenous Pro can mediate the response of jasmonate signals-related genes in rice seedlings under Cr(VI) stress, which further affects the expression of downstream genes.

### Expression of genes activated in the MYB-bHLH-WDR complex in rice seedlings

#### Gene expression at EC_20_ of Cr(VI)

In rice roots, relatively higher expression of *OsbHLH6*, *OsbHLH008*, *OsbHLH010*, *OsbHLH014*, *OsMYB55*, and *OsC1-Myb* was detected from “Pro + Cr(VI)” treatments than that from Cr(VI) treatments (*p* < 0.05), while in rice shoots, relatively higher expression of *OsbHLH6*, *OsbHLH010*, *OsbHLH165*, *OsMYB55*, *OsC1-Myb*, and *OsTTG1* was observed from “Pro + Cr(VI)” treatments than that from Cr(VI) treatments (*p* < 0.05; Figures 2A,B).

#### Gene expression at EC_50_ of Cr(VI)

In rice roots, significantly higher expression of *OsbHLH6*, *OsbHLH008*, *OsbHLH014*, *OsMYB55*, and *OsC1-Myb* was detected from “Pro + Cr(VI)” treatments than that from Cr(VI) treatments (*p* < 0.05), while in rice shoots, significantly higher expression of *OsbHLH6*, *OsbHLH010*, and *OsMYB55* was observed from “Pro + Cr(VI)” treatments than that from Cr(VI) treatments (*p* < 0.05; Figures 2A,B).

#### Gene expression at EC_75_ of Cr(VI)

In rice roots, relatively higher expression of *OsbHLH6*, *OsbHLH008*, *OsbHLH010*, *OsbHLH13*, *OsbHLH014*, *OsMYB55*, and *OsC1-Myb* was detected from “Pro + Cr(VI)” treatments than that from Cr(VI) treatments (*p* < 0.05), while in rice shoots, relatively higher expression of *OsbHLH6*, *OsbHLH010*, *OsbHLH15*, *OsMYB55*, and *OsMYB55* was observed from “Pro + Cr(VI)” treatments than that from Cr(VI) treatments (*p* < 0.05; Figures 2A,B).

These results indicated that the expression of MYB-bHLH-WDR complex-related genes in rice tissues is highly dependent on the EC concentrations of Cr(VI). Exogenous Pro can positively regulate the expression of genes functioning in the MYB-bHLH-WDR complex in rice plants under Cr(VI) stress and affects anthocyanidin synthesis.

### Expression of genes responsible for anthocyanidin synthesis in rice seedlings

#### Gene expression at EC_20_ of Cr(VI)

In rice roots, *OsASN1* and *OsASN2* under “Pro + Cr(VI)” treatments were significantly (*p* < 0.05) upregulated, compared with Cr(VI) treatments, while in rice shoots, higher expression of *OsDFR*, *OsASN1*, and *OsASN2* under “Pro + Cr(VI)” treatments was observed (*p* < 0.05), rather than Cr(VI) treatments (Figures 2A,B).

#### Gene expression at EC_50_ of Cr(VI)

In rice roots, higher expression of *OsASN1* and *OsASN2* under “Pro + Cr(VI)” treatments was detected (*p* < 0.05), rather than Cr(VI) treatments, while in rice shoots, higher expression of *OsDFR* and *OsASN2* under “Pro + Cr(VI)” treatments was detected (*p* < 0.05), rather than Cr(VI) treatments (Figures 2A,B).

#### Gene expression at EC_75_ of Cr(VI)

No significant difference was observed in the expression of genes responsible for anthocyanidin synthesis in both rice tissues at all treatments (Figures 2A,B). These results suggested that the regulatory effects of exogenous Pro in rice seedlings were weak under EC_75_ of Cr(VI).

### The gene expression variation factors at different ECs of Cr(VI)

**Figure 3:**
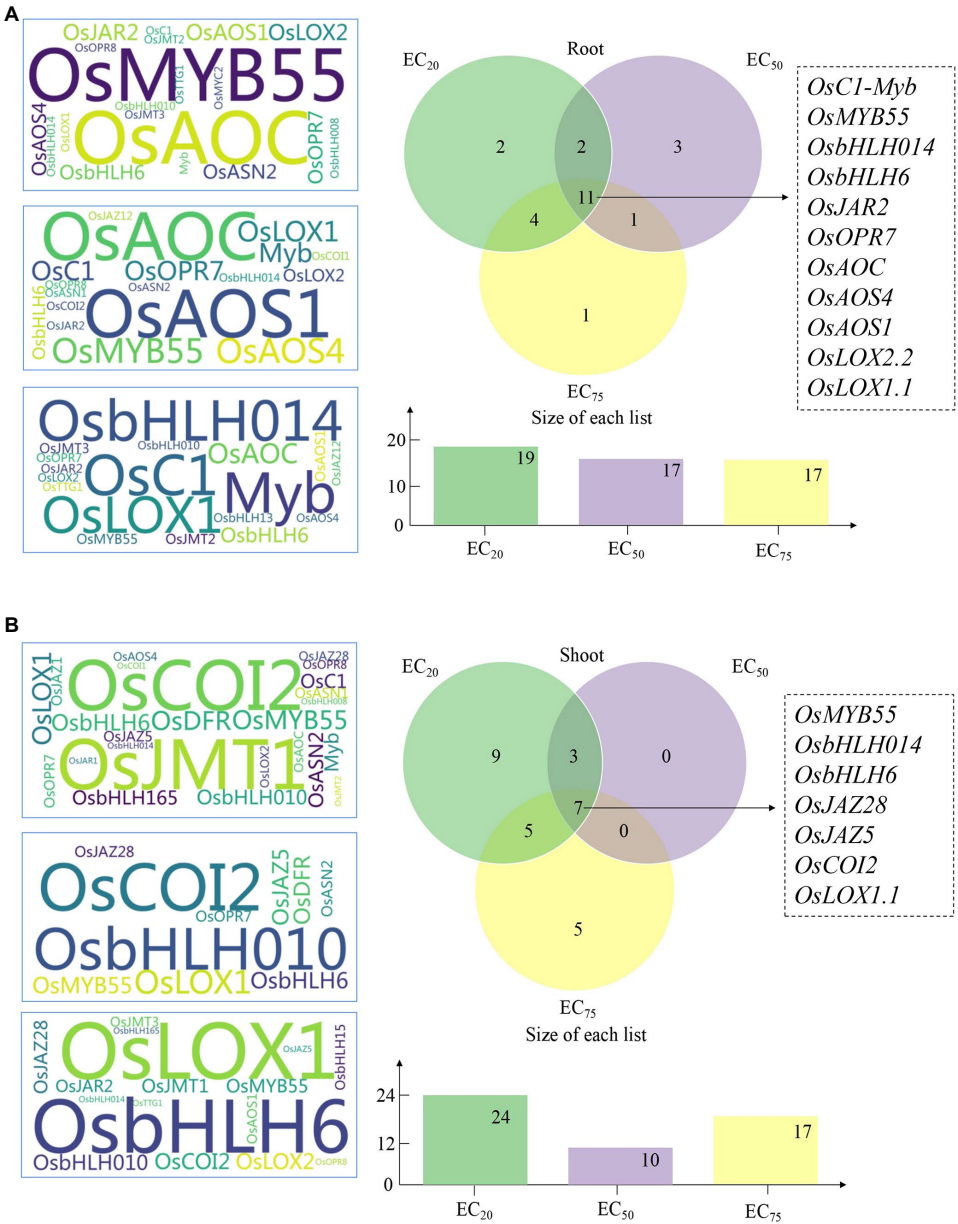
The genetic expression variation factors (*GEVFs*) between Cr(VI) treatments and Pro + Cr treatments in rice seedlings. **(A)** Left: the *GEVFs* of “promoting genes” in root tissues at three ECs of Cr(VI; ^*^the larger size of gene symbol indicates a higher value of *GEVFs*); Right: the Venn diagram of these “promoting genes” at three ECs of Cr(VI). **(B)** Left: the *GEVFs* of “promoting genes” in shoot tissues at three ECs of Cr(VI; ^*^the larger size of gene symbol indicates a higher value of *GEVFs*); Right: the Venn diagram of these “promoting genes” at three ECs of Cr(VI).

Influence of exogenous Pro on the expression of genes responsible for JA synthesis (Module 1), JA signaling pathway (Module 2), MYB-bHLH-WDR complex (Module 3), and anthocyanidin synthesis (Module 4) at different ECs of Cr(VI) was estimated by the *GEVFs* (Figure 3), and the detailed information was shown in Supplementary Table S2. Venn diagram showed that *OsLOX1.1, OsLOX2.2, OsAOS1, OsAOS4, OsAOC, OsOPR7,* and *OsJAR2* (Module 1) and *OsbHLH6, OsbHLH014, OsMYB55,* and *OsC1-Myb,* (Module 3) are the common “promoting genes” detected at three ECs of Cr(VI) in rice roots (Figure 3A), suggesting that exogenous Pro had a stronger impact on the JA synthesis and the MYB-bHLH-WDR complex than others in rice seedlings at three treatment concentrations of Cr(VI). Additionally, we noticed that the number of “promoting genes” in both rice tissues decreased with the increasing Cr(VI) concentrations, indicating the higher Cr(VI) concentration the less regulation effect of exogenous Pro. Herein, five “promoting genes” with the highest *GEVF* values in rice tissues at different ECs of Cr(VI) were listed. In the root part, genes *OsMYB55* (Module 3)*, OsAOC* (Module 1)*, OsAOS1* (Module 1)*, OsJAR2* (Module 1), and *OsLOX2.2* (Module 1) showed higher *GEVFs* values at EC_20_ of Cr(VI); *OsAOS1* (Module 1)*, OsAOC* (Module 1)*, OsMYB55* (Module 3)*, OsAOS4* (Module 1), and *OsLOX1.1* (Module 1) displayed the higher *GEVFs* values at EC_50_ of Cr(VI); *OsbHLH014* (Module 3)*, OsC1-Myb* (Module 3)*, OsLOX1.1* (Module 1)*, OsAOC* (Module 1), and *OsbHLH6* (Module 3) presented higher *GEVFs* values at EC_75_ of Cr(VI).

In rice shoots, Venn diagram showed that *OsLOX1.1* (Module 1), *OsJAZ28*, *OsJAZ5*, and *OsCOI2* (Module 2), and *OsbHLH6*, *OsbHLH014*, *OsJAZ28*, and *OsMYB55* (Module 3) are the common “promoting genes” detected at three ECs of Cr(VI; Figure 3B). Also, *OsJMT1* (Module 1), *OsCOI2* (Module 2), *OsDFR* (Module 4), *OsMYB55* (Module 3), and *OsLOX1.1* (Module 1) showed the higher *GEVFs* values at EC_20_ of Cr(VI); *OsCOI2* (Module 2), *OsbHLH010* (Module 3), *OsLOX1.1* (Module 1), *OsMYB55* (Module 2), and *OsJAZ5* (Module 2) displayed the higher *GEVFs* values at EC_50_ of Cr(VI); *OsLOX1.1* (Module 1), *OsbHLH6* (Module 3), *OsCOI2* (Module 2), *OsLOX2.2* (Module 2), and *OsbHLH010* (Module 3) presented higher *GEVFs* values at EC_75_ of Cr(VI).

### Gene interaction and regulation network in rice plants

**Figure 4:**
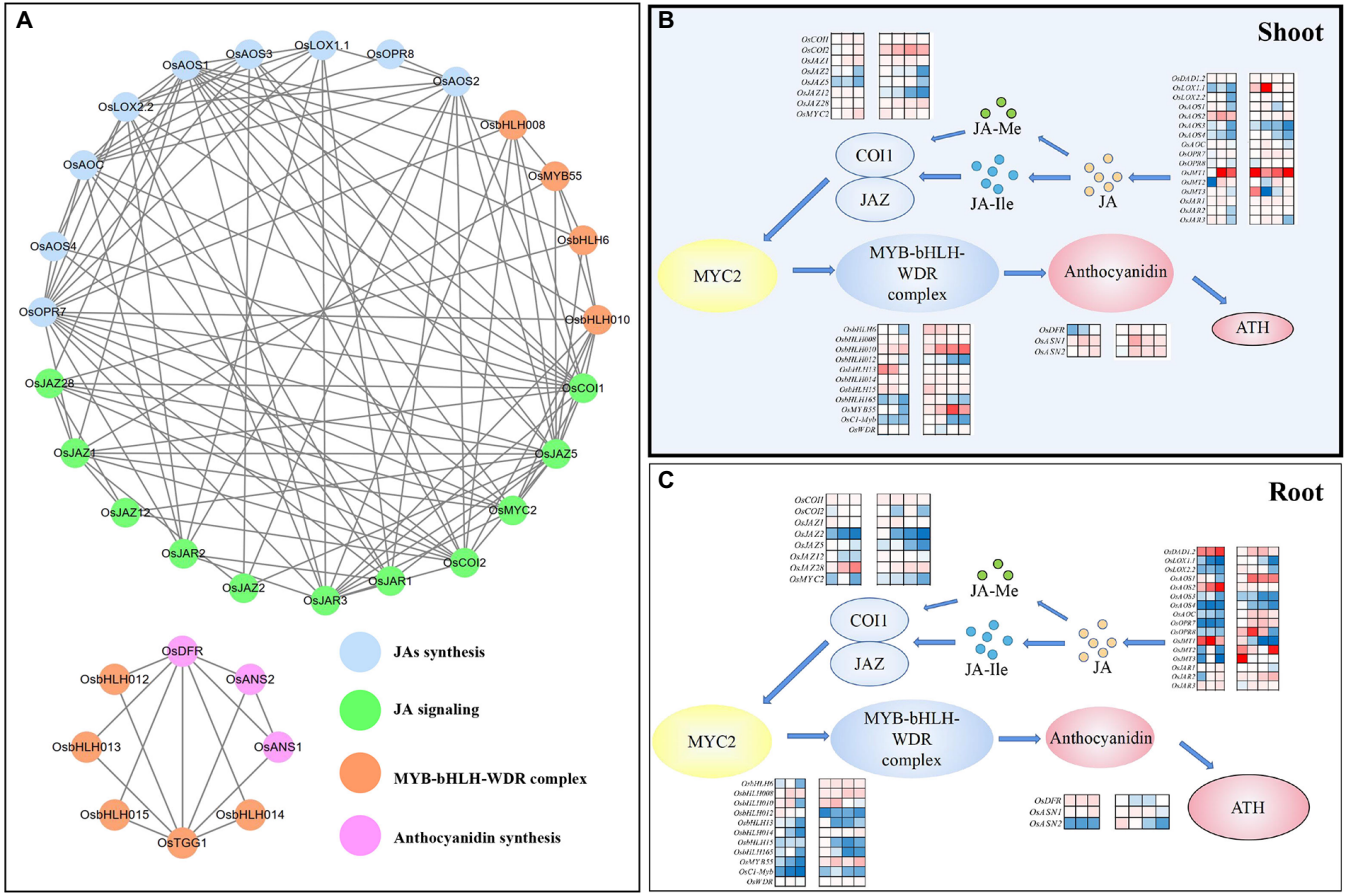
The schematic representation of the Pro-mediated regulation network of JA synthesis, JA signaling, MYB-bHLH-WDR complex, and anthocyanidin synthesis in rice seedlings under Cr stress. **(A)** The genetic interaction network in rice seedlings; **(B)** Heatmap presentation of Pro-mediated regulation of JA synthesis, JA signaling, MYB-bHLH-WDR complex, and anthocyanidin synthesis in rice shoots under Cr stress; **(C)** Heatmap presentation of Pro-mediated regulation of JA synthesis, JA signaling, MYB-bHLH-WDR complex, and anthocyanidin synthesis in rice roots under Cr stress.

The interaction network of genes selected in this study is shown in Figure 4A. Based on the analysis of the interaction network, genes of *OsAOS1, OsAOC, OsJAZ5, OsAOS2, OsAOS3, OsAOS4, OsCOI1, OsJAR3, OsCOI2, OsLOX1.1, OsJAZ1, OsJAR1, OsDFR, OsJAR3, OsJAR1,* and *OsJAR2* had higher combined scores (>0.9), suggesting higher interactive capacities. In addition, heatmap presentation of Pro-mediated response of genes activated in Modules 1 to 4 in rice tissues in response to Cr(VI) exposure was mapped (Figures 4B,C), suggesting that exogenous Pro-mediated regulation was tissue-specific and a more positive effect was detected in shoots than roots under Cr(VI) exposure.

## Discussion

Plant growth and development are highly coordinated with species and levels of hormones in their cells. Fluctuation and imbalance of phytohormone levels in plant tissues are crucial to integrating with signaling modulation and stress responses (Sytar et al., 2019). A previous study indicated that Cr exposure evoked a series of complex hormone responses in plants and disturbed the physiological and molecular processes (Asad et al., 2018). Being a plant nutritional regulator, Pro is considered to be a useful “alleviator” that curtails the adverse impact induced by Cr pollution on rice plants (Gomes et al., 2017; Yang et al., 2021). In this study, we focused on the effects of exogenous Pro on the resistance and tolerance of rice plants to different ECs of Cr(VI), judged by the repression of ATH accumulation through regulating jasmonate signals and the MYB-bHLH-WDR complex. Therefore, the possible regulation mechanisms of exogenous Pro on these biochemical and molecular processes in rice under Cr(VI) stress is discussed accordingly.

### Exogenous proline mediates jasmonate signals in rice under Cr(VI) stress

Synthesis of JA in plants occurs in the plastid, peroxisome, and cytoplasm (Raza et al., 2021). Various stresses can activate phospholipases in the plastid membrane and promote the conversion of linolenic acid (a precursor for JA synthesis) into OPDA in the presence of enzymes LOX, AOS, and AOC (Ruan et al., 2019). After that, JA is synthesized from OPDA by the OPR and three cycles of β-oxidation (Ruan et al., 2019). Results from the current study demonstrated that Cr(VI) exposure at three EC concentrations significantly increased the content of JA in rice shoots. Also, JA synthesis-related genes (i.e., *OsDAD1.2*, *OsAOS2*, *OsAOC,* and *OsOPR7*) showed upregulation in rice shoots under different ECs of Cr(VI) exposure. These results indicated that JA synthesis in rice plants is positive in response to Cr(VI) stress. A recent study suggested that Cd exposure significantly regulated the expression of JA synthesis-related genes in *A. thaliana*, with an increase in endogenous JA levels in root parts (Lei et al., 2020). Also, previous studies reported that exogenous JA can alleviate the detrimental effects of toxic metals (e.g., Ni, Cu, and Pb) on plants by regulating antioxidants (Sirhindi et al., 2016), increasing chlorophyll content (Dai et al., 2020), and inducing synthesis of secondary metabolites (e.g., total phenols, polyphenols, and flavonoids; Bali et al., 2019).

In the cytoplasm, JA can conjugate with isoleucine (Ile) to form JA-Ile, while it is also converted into JA-Me through JA carboxylmethyl transferase (Ali and Baek, 2020). Both compounds initiate the JA signaling pathway. In this study, we noticed that the content of JA-Ile in rice shoots increased with enhancing Cr(VI) concentrations, and upregulation of JA-Ile synthesis-related genes (i.e., *OsJAR1, OsJAR2,* and *OsJAR3*) was detected in rice shoots at three ECs of Cr(VI) exposure. In addition, genes involved in JA-Me synthesis (i.e., *OsJMT1* and *OsJMT2*) were positively activated in shoot tissues at EC_50_ and EC_75_ of Cr(VI) exposure. These results suggested that Cr(VI) exposure at EC_50_ and EC_75_ stimulated the synthesis of JA-Ile and JA-Me in rice shoots, while JA-Ile synthesis at EC_20_ of Cr(VI) exposure was more sensitive than JA-Me synthesis. It is known that JA-Ile as a bioactive compound in plant cells is always kept at a very low level under normal conditions (Fonseca et al., 2009), in which genes responsible for the JA signaling pathway remained in the inactivated state (Ali and Baek, 2020). When plants suffered unfavorable conditions, the trade-off between epimerization of JA into JA-Ile and accumulation of JA-Ile became a mandatory routine in the cytoplasm of aerial parts, afterwards, JA signaling pathway was activated by JA-Ile accumulated (Truman et al., 2007; Li et al., 2017). Herein, the expression of JA signaling pathway-related genes (i.e., *OsCOI1, OsCOI2, OsJAZ1, OsJAZ12, OsJAZ28*) was significantly upregulated in rice shoots after Cr(VI) exposure, suggesting that JA-Ile can positively bind with the COI and JAZ proteins and activated their capacity of signal transduction (Wasternack and Hause, 2013; Wasternack and Song, 2017; Raza et al., 2021). In addition, a significant increase in the expression of *OsMYC2* was observed in rice shoots after Cr(VI) exposure. It is known that *OsMYC2* is highly involved in the JA signaling pathway in rice through binding to the JAZ and COI proteins, thereby stimulating the expression of downstream JA response genes (Thines et al., 2007).

Proline functioning in plants as an osmoprotectant, ROS scavenger, and membrane stabilizer has been studied extensively (Hoque et al., 2007; Moustakas et al., 2011; Yu et al., 2017; Zhang et al., 2021). Herein, exogenous Pro-mediated regulation on JAs synthesis and signaling pathway was first documented in rice plants under Cr(VI) stress. Endogenous level of JA and JA-Ile in rice shoots under “Pro + Cr(VI)” treatments was higher than that of Cr(VI) treatments, suggesting that exogenous Pro promoted the synthesis of JA and JA-Ile in rice plants under Cr(VI) stress. PCR analysis also showed a positive role of exogenous Pro on the expression of JA synthesis and signaling pathway-related genes in shoots of rice seedlings at different ECs of Cr(VI; Figure 4B). Calculation of *GEVFs* indicated that exogenous Pro-mediated regulation was tissue-specific. A stronger regulation impact of exogenous Pro on the JA synthesis was observed in roots, while both JA synthesis and signal pathway in shoots were regulated by exogenous Pro applied. Additionally, Venn diagram showed that *OsLOX1.1* (Module 1) was the common promoting gene regulating JA synthesis in both rice tissues in response to Cr(VI) stress. An indirect link between Pro and plant hormone was observed in plants during Cr stress (Alvarez et al., 2022), wherein Pro regulates the level of other signaling molecules (e.g., ROS, H_2_S, and NO; Verbruggen and Hermans, 2008), thereby affecting the response pattern of JA and JA-Ile content in plants.

### Exogenous proline activates the MYB-BHLH-WDR complex in rice under Cr(VI) stress

A previous study indicated that the MYB-bHLH-WDR complex in plants is a valve between the JA signal pathway and the downstream JA response genes in responses to various stresses (Qi et al., 2011). In this current study, more genes activated in MYB-bHLH-WDR complex were upregulated in “Pro + Cr(VI)” treatments than Cr(VI) treatments, and higher expression of genes was observed in “Pro + Cr(VI)” treatments than Cr(VI) treatments (Figure 4B). Additionally, calculation of *GEVFs* indicated that *OsbHLH6, OsbHLH014,* and *OsMYB55* are the common promoting genes in both rice tissues regulating the MYB-bHLH-WDR complex under Cr(VI) stress. We also noticed that the regulation role of exogenous Pro on the MYB-bHLH-WDR complex in rice shoots was highly dependent on Cr(VI) concentrations, in which expression of *OsMYB55*, *OsbHLH010* and *OsMYB55*, and *OsbHLH6* and *OsbHLH010* was highly activated by exogenous Pro at EC_20_, EC_50_, and EC_75_ of Cr(VI), respectively. These results indicated different regulation strategies of the MYB-bHLH-WDR complex mediated by exogenous Pro in rice plants under Cr(VI) stress.

### Exogenous proline inhibits ATH accumulation in rice under Cr(VI) stress

It is known that anthocyanidin can possess great potential for alleviating photooxidation as a shelter to protect the damaging impact of excess light absorbed on chloroplasts (Zhang et al., 2002; Zhang and Becker, 2015), and serves as a ROS scavenger to diminish oxidative burst in plants (Luo et al., 2016). Anthocyanin is a byproduct formed by the glycosidic bond between anthocyanidin and sugars (Ling et al., 2021), which is frequently detected in plants under unfavorable conditions (Ling et al., 2021). Herein, although expression of anthocyanidin synthesis-related genes (*OsDFR, OsASN1,* and *OsASN2*) in rice shoots was upregulated at all treatments, the glycosylation of anthocyanidin into ATH in rice plants was also detected. More accumulation of ATH was observed in rice plants with enhancing Cr(VI) concentrations, suggesting that Cr(VI) exposure can simulate the glycosylation of anthocyanidin and enhance the accumulation of ATH in plants. However, a reduction in the endogenous level of ATH in Cr(VI)-treated rice seedlings was observed due to the application of exogenous Pro, suggesting that exogenous Pro can effectively repress the glycosylation of anthocyanidin and decrease ATH accumulation in rice plants under Cr(VI) stress.

### Environmental implications

Chromium pollution from industrial processes seriously influences the safety of the ecosystem. In some cases, a higher level of Cr can enter the agricultural systems and threaten the yield, quality, and food safety of crops. Recently, plant-derived growth regulators (PDGRs) have been used to minimize the adverse effects imposed by heavy metals, due to their availability (Yang et al., 2021). Compared with other chemical reagents used, PDGRs not only improve the resistance capacity of plants against heavy metal stresses but also alter the distribution pattern of heavy metals in plant tissues (Hao et al., 2012). Due to their different chemical properties and occurrence in plant cells, the regulatory mechanisms of PDGRs in alleviating phytotoxicity caused by heavy metals is different. For instance, exogenous H_2_S can manage Cr(VI) toxicity in wheat, rice, and bean through the ethylene signaling, sulfur assimilation, and ascorbate-glutathione cycle (Husain et al., 2020, 2021, 2022; Singh et al., 2022a,b). Also, exogenous amino acids (e.g., Pro, gamma-aminobutyric acid, and glutamate) can minimize heavy metal toxicity in rice, tomato, and brinjal (Jiang et al., 2020; Suhel et al., 2022; Zhang et al., 2022) by mediating the transcription factors and the antioxidant systems. Additionally, exogenous plant hormones (e.g., JA, auxin, kinetin, and melatonin) can regulate metal uptake, photosynthetic pigments, and antioxidant systems to enhance stress resistance (López et al., 2007; Wang et al., 2015; Dai et al., 2020; Hodžić et al., 2021). In this study, we displayed the effects of exogenous Pro on ATH accumulation through regulating Jasmonate signals and MYB-bHLH-WDR complex and protecting rice plants from Cr(VI) stress.

## Conclusion

This study presents the latest evidence to update the positive role of exogenous Pro on regulating jasmonate signals and activating the MYB-bHLH-WDR complex to repress ATH accumulation in rice plants under Cr(VI) stress. New findings include: (1) exogenous Pro promotes an endogenous level of JA and JA-Ile in rice tissues under Cr(VI) stress; (2) increment in the level of jasmonates triggers the expression of genes activated in the JA signaling pathway; (3) the activation of MYB-bHLH-WDR complex inhibits the glycosylation of anthocyanidin in rice tissue and decreases ATH accumulation in rice plants; (4) exogenous Pro-mediated regulation on jasmonate signals was tissue-specific under Cr(VI) stress and a more positive effect was detected in rice shoots rather than roots.

## Data availability statement

The original contributions presented in the study are included in the article/Supplementary material, further inquiries can be directed to the corresponding author.

## Author contributions

X-ZY: conceptualization, methodology, supervision, writing-reviewing and editing, and funding acquisition. QZ: investigation. Y-XF: writing-original draft preparation and visualization. PT: investigation, data analysis, visualization, and software. Y-JL: data analysis, visualization, and software. All authors contributed to the article and approved the submitted version.

## Funding

This work was financially supported by the Natural Science Foundation of Guangxi (no. 2018GXNSFDA281024).

## Conflict of interest

The authors declare that the research was conducted in the absence of any commercial or financial relationships that could be construed as a potential conflict of interest.

## Publisher’s note

All claims expressed in this article are solely those of the authors and do not necessarily represent those of their affiliated organizations, or those of the publisher, the editors and the reviewers. Any product that may be evaluated in this article, or claim that may be made by its manufacturer, is not guaranteed or endorsed by the publisher.
