# Supplementary material for: Proline-mediated regulation on jasmonate signals repressed anthocyanin accumulation through the MYB-bHLH-WDR complex in rice under chromium exposure
Source: Front Plant Sci. 2022 Aug 2;13:953398. doi: 10.3389/fpls.2022.953398 (PMC9379311; doi:10.3389/fpls.2022.953398)
Supplement: Supplementary file 1 [file Table_1.DOC]

***Supporting information for***

**Proline-mediated** **regulation on jasmonate signals repressed anthocyanin accumulation through the** **MYB-bHLH-WDR complex in rice under chromium exposure**

Qing Zhang^1^, Yu-Xi Feng^1^, Peng Tian, Yu-Juan Lin, Xiao-Zhang Yu^*^

College of Environmental Science & Engineering, Guilin University of Technology, Guilin 541004, People's Republic of China

**^*^Corresponding author**

Prof. Dr. Xiao-Zhang Yu. ORCID iD: 0000-0001-7846-5017

Phone: +86 7735897016. E-mail: [xzyu@glut.edu.cn](mailto:xzyu@glut.edu.cn)

^1^ The authors contribute equally to this work.

**Supporting information M1.**

**Measurement of jasmonates.** *Extraction and Purification of tissues samples:* 100.0 mg of the powdery tissue samples was homogenized and extracted for 12 h in 1.0 mL of methanol solution (0.2% formic acid). The process was isolated from light and kept at 4°C. Next, the mixture was centrifuged at 14,000 g for 10 min at 4°C. The supernatant was taken and evaporated with N_2_. Subsequently, the residue was resuspended in 0.2 mL of methanol solution (50%, v/v), and then centrifuged at 14,000 g for 10 min at 4°C. The supernatant was collected for phytohormone analysis.

*UPLC-MS analysis*: The mobile phase buffer included a combination of buffer A (0.04% formic acid in water) and buffer B (0.04% formic acid in acetonitrile). The injection volume was 4.0 μL at a flow rate of 0.4 μL/min, automatic sampler temperature was 4°C, and the column temperature was 45°C. The linear gradient was as follows: 2%→98% B (v/v) for 10 min; 98%→2% B (v/v) to 10.1 min; and hold at 2% B to 13 min.

The MS system (5500 QTRAP System, AB SCIEX) equipped with an electrospray ionization source was operated in negative ionization and multiple reaction monitoring modes. The running conditions of 5500 QTRAP were as follows: source temperature, 500 °C; ion Source Gas 1, 45 psi; ion Source Gas 2, 45 psi; Curtain gas, 30 psi; and ionSapray Voltage Floating, -4500 V.


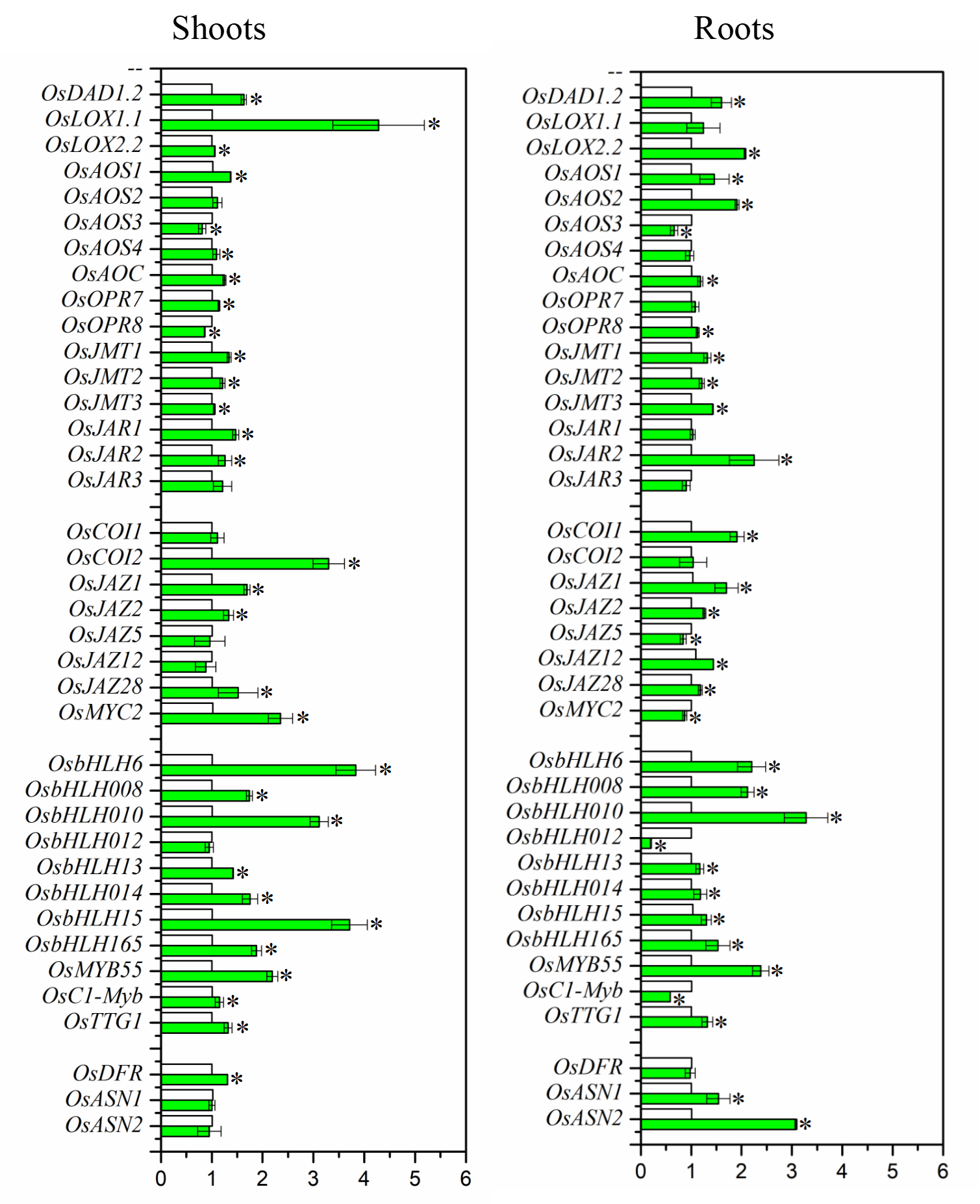


**Fig. S1** Gene expression of module 1-4 in rice seedlings at 0 mg Cr/L treatment (i.e., EC_0_) with or without exogenous Pro supplied.

**Table S1** Primer sequences of JA and anthocyanin synthesis genes and related transcription factor genes

| **Gene name** | **Locus identifier** | **Primer sequences（5'-3'）** | **Amplicon size （bp）** |
| --- | --- | --- | --- |
| Phospholipase A1 （DAD） | | | |
| *OsDAD1.2* | LOC_Os08g04800.1 | F-AGGCGTACATCCACCTCGT  R-AGTGCACGCACTCCAAGAC | 201 |
| Lipoxygenase （LOX） | | | |
| *OsLOX1.1* | LOC_Os03g52860.1 | F-TACAAGGACTGGAGCTTCGC  R-CGTCGTACTCCTTCGTTCCC | 463 |
| *OsLOX2.2* | LOC_Os12g37260.1 | F-CAGTACCAGACCACCCTCGT  R-TGCTCATCATCCTCTCGTTG | 154 |
| Allene oxide synthase （AOS） | | | |
| *OsAOS1* | LOC_Os03g55800.1 | F-ACGACTACGGGTACGAGGTG  R-GAGCACGACGAAGTCCTTG | 227 |
| *OsAOS2* | LOC_Os03g12500.1 | F-CAAGAAGGGGGAGATGCTGT  R-TAGGGTTCTCCGTCTCTCGC | 167 |
| *OsAOS3* | LOC_Os02g12680.1 | F-CTCCCCATACGCGAGATAC  R-CTGGAAGTAGAAGTAGTCGAGG | 256 |
| *OsAOS4* | LOC_Os02g12690.1 | F-GTGGCTCATCTTCCAGCTTC  R-AACACGAGGTTGTGGAGGAG | 222 |
| Allene oxide cyclase （AOC） | | | |
| *OsAOC* | LOC_Os03g32314.1 | F-GCTACGAGGCCATCTACAGC  R-TGCCCTTGAGGTAGAAGGTG | 195 |
| 12-oxophytodienoate reductase （OPR） | | | |
| *OsOPR7* | LOC_Os08g35740.1 | F-TGACCGCACTGACGAGTATG  R-ATGCCGAGCTTAATGGGGTC | 168 |
| *OsOPR8* | LOC_Os02g35310.1 | F-GGACTCAGACCCAGATGCAC  R-GACTGAGAAAAGGGTAATCGGTG | 371 |
| Jasmonyl-L-isoleucine synthase （JAR） | | | |
| *OsJAR1* | LOC_Os05g50890.1 | F-ACCTCCAGTTGGCTGTTGAG  R-CCTTGCTGTTTGATGGGTTC | 354 |
| *OsJAR2* | LOC_Os01g12160.1 | F-CGCACTACCTCTCCCTCGGCG  R-GCGTCTTTGGTTTGCAATTCG | 220 |
| *OsJAR3* | LOC_Os11g08340.1 | F-CAAGCGTCTCCTGTTCAATGAC  R-GCGTAGCTGGCATGGAAGAA | 66 |
| JA carboxylmethyl transferase (JMT) | | | |
| *OsJMT1* | LOC_Os01g50610.1 | F-CGTTCAGGATGGACTACGTG  R-CAATCTGGACGCTCTTCACC | 219 |
| *OsJMT2* | LOC_Os06g22440.1 | F-GCACGGCAGTTCAGGAAGGA  R-TTCGGAGGCGAGATCAACAGAT | 120 |
| *OsJMT3* | LOC_Os06g20920.1 | F-GGATGCTTATGCTCGCCAGTTC  R-CTACCACCTGCCATGCCTGAT | 155 |
| Coronatine insensitive 1 （COI） | | | |
| *OsCOI1* | LOC_Os01g63420.1 | F-GATGCCCTCCCTGAGATACA  R-GCAGTCAGACCTCCTTCCAG | 214 |
| *OsCOI2* | LOC_Os03g15880.4 | F-TTAACGATTTCCGGCTTGTC  R-GCAGTCCTTGGTCAGACTCC | 228 |
| Jasmonate ZIM-domain protein （JAZ） | | | |
| *OsJAZ1* | LOC_Os04g55920.1 | F-TCCACCTAATCCTTCGCAAC  R-GAGACTGCTGCTTGGGTAGC | 201 |
| *OsJAZ2* | LOC_Os07g05830.1 | F-GGGGACGACAGTACAAGTGC  R-CGTCCCTACCCTGTATTTGC | 193 |
| *OsJAZ5* | LOC_Os04g32480.1 | F-TGTGTGCTTCACAGTATTTCG  R-GGACAGAAGCAGCTCCAATG | 282 |
| *OsJAZ12* | LOC_Os02g49970.1 | F-TGCCTGGAACGACTGAACAG  R-TGGGACGTTGTCGAACTTCAC | 219 |
| *OsJAZ28* | LOC_Os09g26780.1 | F-TGGAGAGAAGGCTCAACAGC  R-CACCGTTAGCTTGCTTGGAC | 305 |
| Myelocytomatosis proteins （MYC） | | | |
| *OsMYC2* | LOC_Os10g42430.1 | F-ACCGGCGAGATCCTAAACTT  R-GGACCTCTTCTGGTTGTTGC | 177 |
| Dihydroflavonol-4-reductase （DFR） | | | |
| *OsDFR* | LOC_Os01g44260.1 | F- CACGACGCCACCATCCAC  R-CGGTCCCCCTCCCCAGTA | 310 |
| Anthocyanidin synthase （ANS） | | | |
| *OsANS1* | LOC_Os01g27490.1 | F- GCCTCTCCTGGGTCGTCT  R- TGCGGCATTGTTGTCTTG | 176 |
| *OsANS2* | LOC_Os06g42130.1 | F-GCCAGCGGGAAGAGGGAGT  R- GCCGTTGTGGAGGATGAAG | 357 |
| Basic helix-loop-helix transcription activator （bHLH） | | | |
| *OsbHLH6* | LOC_Os04g23550.1 | F-GGAGCGGACGAAGAAGGTGAAG  R-GGAGGTGATGTTGGCGGTGATG | 235 |
| *OsbHLH008* | LOC_Os01g13460.1 | F-GGACATTCAGGTGGTTCAGGAT  R-CGTGCCGTTGTTGCTTGTG | 142 |
| *OsbHLH010* | LOC_Os01g50940.1 | F-TACGCACCGTCGTCTTCCT  R-GCCTTGACCACCTCCTTCTTG | 268 |
| *OsbHLH012* | LOC_Os01g39480.1 | F-TGCTCATCGACGACCTCTTCCT  R-GGCGGTGGCTGCTTGTTGTT | 110 |
| *OsbHLH013* | LOC_Os04g47080.1 | F-CGGCGATGACGACTCAAGGAAG  R-GCGATGGTTGGCTGCTGGATT | 213 |
| *OsbHLH014* | LOC_Os11g15210.1 | F-GCCTGGATGAACAATGCTGAC  R-CGATGGTTGGCTGCTGGAT | 246 |
| *OsbHLH015* | LOC_Os04g47040.1 | F-CAGCAGCGAGCCATCACATCAG  R-CCAACTCCTTCCATCGGCACTG | 194 |
| *OsbHLH165* | LOC_Os01g39580.1 | F-TGTCGGTGGAAGGAGCTGATG  R-GCGGCAGAACTGGCATACTTAG | 140 |
| R2R3-MYB transcription factor（MYB） | | | |
| *OsMYB55* | LOC_Os05g48010.1 | F-TCATCTGCTCGCTCTACAACTC  R-GGTACATGGTGGTGGTGGTG | 276 |
| *OsC1-Myb* | LOC_Os06g10350.1 | F-CGTGCTTGCCTCCTACATC  R-GTTAGGCCGGAGATAGTTGAG | 124 |
| Transparent testa glabra1 | | | |
| *OsTTG1* | LOC_Os02g45810.1 | F-CATCTTCGCCTCCGTCTCAG，R-GCCATGTAGTGGAAGTCATAGC | 147 |
| Glyceraldehyde-3-phosphate dehydrogenase （GAPDH） | | | |
| *OsGAPDH1* | LOC_Os08g03290.1 | F-GACAGCAGGTCGAGCATCTTC  R-CAGGCGACAAGCTTGACAAAG | 74 |

**Table S2** The gene expression variation factors at different ECs of Cr(VI).

| Gene name | Shoot | | |  | Root | | |
| --- | --- | --- | --- | --- | --- | --- | --- |
|  | EC_20_ | EC_50_ | EC_75_ |  | EC_20_ | EC_50_ | EC_75_ |
| *OsASN2* | 204.70 | 30.60 | 1.58 |  | 214.27 | 93.38 | -9.47 |
| *OsASN1* | 157.50 | -22.72 | -12.40 |  | 19.14 | 43.98 | -64.47 |
| *OsDFR* | 392.63 | 82.28 | 18.13 |  | -69.02 | -70.55 | -65.67 |
| *OsTTG1* | -34.35 | -5.79 | 35.34 |  | 29.70 | 5.56 | 35.71 |
| *OsC1-Myb* | 165.02 | -22.37 | -31.02 |  | 128.05 | 267.71 | 671.02 |
| *OsMYB55* | 268.51 | 1039.38 | 1217.70 |  | 473.06 | 480.41 | 2769.29 |
| *OsbHLH165* | 194.55 | 0.13 | 53.66 |  | -1.42 | -76.74 | -20.90 |
| *OsbHLH15* | -34.02 | -38.76 | 72.51 |  | -31.88 | -65.89 | -43.54 |
| *OsbHLH014* | 31.47 | -9.99 | 27.93 |  | 35.00 | 147.02 | 819.74 |
| *OsbHLH13* | -78.13 | -70.99 | -8.10 |  | -59.68 | -51.70 | 91.23 |
| *OsbHLH012* | -34.60 | -69.72 | -59.01 |  | -80.50 | -69.30 | -71.32 |
| *OsbHLH010* | 243.64 | 160.03 | 123.96 |  | 143.92 | -67.19 | 85.81 |
| *OsbHLH008* | 29.94 | -13.91 | -7.38 |  | 77.85 | 14.09 | 20.67 |
| *OsbHLH6* | 251.27 | 82.39 | 181.71 |  | 217.29 | 158.42 | 316.60 |
| *OsMYC2* | 17.47 | 8.22 | -56.31 |  | 34.75 | -26.19 | -1.13 |
| *OsJAZ28* | 77.30 | 49.05 | 113.84 |  | 14.65 | -39.90 | -61.35 |
| *OsJAZ12* | -48.60 | -74.44 | -81.28 |  | 16.83 | 42.50 | 25.95 |
| *OsJAZ5* | 84.30 | 94.52 | 26.62 |  | -17.68 | -47.71 | -66.57 |
| *OsJAZ2* | -11.16 | -8.43 | -45.82 |  | -19.08 | 3.95 | -43.75 |
| *OsJAZ1* | 83.44 | -7.20 | -36.16 |  | 21.46 | -13.92 | 12.46 |
| *OsCOI2* | 411.22 | 359.56 | 158.91 |  | -26.90 | 32.98 | -44.66 |
| *OsCOI1* | 26.56 | -5.10 | -32.77 |  | 20.89 | 30.40 | 9.39 |
| *OsJAR3* | 4.01 | 0.72 | -20.11 |  | -17.95 | 12.51 | -40.69 |
| *OsJAR2* | 22.20 | 5.49 | 91.19 |  | 271.56 | 126.81 | 113.09 |
| *OsJAR1* | 27.12 | -2.64 | 24.44 |  | -3.23 | -25.28 | -59.45 |
| *OsJMT3* | -77.80 | -34.71 | 74.63 |  | 88.05 | -4.92 | 172.24 |
| *OsJMT2* | 26.98 | 2.79 | -5.27 |  | 68.99 | -33.39 | 186.33 |
| *OsJMT1* | 431.21 | -6.95 | 99.09 |  | -88.75 | -97.18 | -92.26 |
| *OsOPR8* | 41.46 | 3.68 | 25.82 |  | 105.06 | 70.97 | -10.72 |
| *OsOPR7* | 126.91 | 60.39 | 9.68 |  | 2117.05 | 3368.84 | 1375.29 |
| *OsAOC* | 60.87 | -20.95 | -11.17 |  | 421.60 | 495.84 | 388.47 |
| *OsAOS4* | 58.93 | 0.59 | -0.61 |  | 168.50 | 434.63 | 56.11 |
| *OsAOS3* | -37.45 | -45.72 | -49.03 |  | -11.19 | -72.83 | -27.93 |
| *OsAOS2* | -57.63 | -72.23 | -44.78 |  | -61.43 | -87.29 | -90.77 |
| *OsAOS1* | 7.79 | -49.42 | 70.11 |  | 299.99 | 538.14 | 1380.54 |
| *OsLOX2.2* | 50.45 | 10.52 | 131.97 |  | 227.22 | 165.87 | 48.07 |
| *OsLOX1.1* | 2605.81 | 109.26 | 330.16 |  | 51.31 | 338.94 | 481.61 |
| *OsDAD1.2* | -10.00 | 2.58 | -2.05 |  | -46.43 | -44.49 | -80.96 |
